# *In vivo* and *in silico* evaluation of antinociceptive activities of seed extract from the *Holarrhena antidysenterica* plant^☆^

**DOI:** 10.1016/j.heliyon.2020.e03962

**Published:** 2020-05-13

**Authors:** Md. Mahbubur Rahman Bhuiyan, N. M. Mahmudul Alam Bhuiya, Md. Nazmul Hasan, Ummey Jannatun Nahar

**Affiliations:** a Department of Pharmacy, University of Development Alternative, Dhaka 1207, Bangladesh; b Department of Pharmacy, Faculty of Life and Earth Sciences, Jagannath University, Dhaka 1100, Bangladesh; c Department of Pharmacy, Faculty of Biological Sciences, University of Chittagong, Chittagong 4331, Bangladesh

**Keywords:** *Holarrhena antidysenterica*, Seed, Peripheral analgesic activity, Central analgesic activity, Molecular docking, ADME, Biological sciences, Plant biology, Pharmaceutical science, Pharmacology, Health informatics, Health profession

## Abstract

The objective of the study was to investigate the analgesic activity of seeds extracted from the *Holarrhena antidysenterica* plant (Family: Apocynaceae). The seeds of *H. antidysenterica* were extracted with pure ethanol and administered to the experimental Swiss albino mice at three different doses (50, 100, and 150 mg/kg body weight) in pain models. Peripheral analgesic activity was evaluated using the acetic acid-induced writhing test, and heat-induced (hot plate and tail immersion test) pain models were applied for central anti-nociceptive activity evaluation. Formalin induced licking test was applied to evaluate both peripheral and central anti-nociceptive activity on mice. Computational studies were performed by Schrödinger Maestro v10.1 for molecular docking and the SwissADME online server for ADME prediction of compounds. In acetic acid-induced writhing test, dose-dependent reduction of writhing response was observed with 43.94% (p < 0.001) writhing inhibition at 150 mg/kg dose compared to standard 60.98% (p < 0.001). 150 mg/kg caused a maximum decrease in licking and biting time in both early and late phases of the formalin-induced licking test (71.2 ± 5.67, p < 0.05, and 36.6 ± 5.62, p < 0.01 respectively). In both tests of central analgesic activity, the extract also showed dose-dependent anti-nociceptive activity. In the hot plate method, the highest %MPE was 67.39 (p < 0.001) at 30 min at 150 mg/kg dose, which was even better than the standard drug. In the case of the tail immersion method, the highest %MPE was 69.84 at a dose of 150 mg/kg at 30 min (p < 0.001). In molecular docking study, Conimine, Conarrhimin, Conessine, and Funtudienine showed the best binding affinities against the COX-1 enzyme. The study indicates that the ethanolic seed extract of *H. antidysenterica* has the strong potentiality of having central analgesic activity and moderate peripheral analgesic activity due to the presence of bioactive compounds in its seeds.

## 1 Introduction

Pain is an unpleasant sensation that acts as a warning of injury or danger in the physiological system and one of the primary reasons patients seek medical care [1]. Generally, primary afferent nociceptors present in the peripheral nervous system and the central nervous system play a vital role in mediating various stimuli that generate feelings of pain by transmitting signals to our brain [2]. Anti-nociceptives or analgesics are chemical substances which reduce the feeling of pain by elevating the threshold of pain to external stimuli often mediated by the prostaglandin pathway [3]. Despite the innovation of newer synthetic agents, analgesics from natural origin is getting more popular, expecting reduced side effect, availability, and low cost. *Holarrhena antidysenterica* (Family: Apocynaceae) is enlisted in classical Ayurveda as an herbal plant for the treatment of Atisara (diarrhea), Jwaratisara (secondary diarrhea), Pravahika (amebiasis), Asra (blood or blood-related disorders), Kustha (skin disorder), and Trisna (thirst) [4]. *H. antidysenterica* is a deciduous tree that is present in different regions of Asia and tropical areas of Africa [5]. In Bangladesh it grows mostly in Dhaka, Chittagong, Cox's Bazar, Sylhet, Dinajpur etc. Locally it is known as Kurchi (Chittagong), Karas (Sylhet), Kutiswar (Dhaka), Indrajab (Dinajpur) etc.

Different parts of the *Holarrhena antidysenterica* plant have been used for the ailment and management of many diseases in traditional medicine. For example, alcoholic, hydromethanolic, aqueous and petroleum ether extracts of its seeds have shown antidiabetic potential [6, 7, 8]. Seeds from this plant have also shown antidiarrheal, diuretic, antiurolithic, antioxidant, antihypertensive, and antibacterial properties [9]. Extracts of barks also have been reported to have a therapeutic effect on inflammatory bowel disease [10]. Some major phytochemicals, namely Conessine, Isoconessine, Conessimine, Conarrhimine, Conimine, Antidysentericine, etc. have already been isolated from the seeds of *H. antidysenterica* [9, 11, 12, 13, 14].

Diversified beneficial properties reported in many types of researches and survey papers have made this plant important to the traditional practitioners as well as the researchers. This study was carried out to investigate the pain-reducing potential of ethanolic extract of *H. antidysenterica* seeds and to establish a pharmacologic basis for using this plant part as herbal medicine. According to Dev, 2006, *H. antidysenterica* was supposed to use in rheumatism and arthritic pain management [15]. Also, to validate the experimental results and to find compounds that could be responsible for in vivo analgesic activity, in silico molecular docking was performed. This computational method effectively identifies the phytochemicals in plant extracts that give pharmacological activity. Thus, the investigation of the analgesic potential of the seeds of the plant in vivo, as well as in the computational model, was the major objective of this research.

## 2 Materials and methods

### 2.1 Chemicals and drugs

Diclofenac sodium and 0.9% NaCl solution were collected from Eskayef Pharmaceuticals Ltd., Bangladesh and Orion Infusion Limited, Tejgaon, Dhaka, Bangladesh, respectively. Gonoshasthaya Pharmaceuticals Limited, Mirjanagar, Asulia, Savar, Dhaka, Bangladesh provided Morphine Sulphate. The local supplier supplied ethanol (Merck, Germany). Rests of the chemicals used in the investigation were analytical grade and collected from the Phytochemical Research Lab, Jagannath University, Dhaka.

### 2.2 Collection of plant material and identification

The seeds of *H. antidysenterica* were collected from Gazipur, Bangladesh. The experts of Bangladesh National Herbarium identified the collected seeds. A voucher specimen was deposited in the Herbarium for future reference (DACB 381570).

### 2.3 Extract preparation

The collected seeds were cleaned to eliminate unwanted materials and then dried in the shade for one and half weeks before being pulverization to produce a powdered sample. Approximately 300 g powder was soaked with two liters of 95% ethanol at 25% w/v. The mixture was kept for seven days with occasional stirring and periodical shaking. The mixture was then filtered through a cotton plug, followed by Whatman No.1 filter paper. Using a rotary evaporator, the filtrate was evaporated to yield the methanol extract of *H. antidysenterica* seeds. The dried crude extract was preserved at 0–4 °C.

### 2.4 Experimental animals

*Swiss albino* mice of both sex having average weight (20–25 g) were used in this study. All mice were collected from Animal Resources Branch of the International Center for Diarrheal Disease Research, Bangladesh (ICDDR, B). Animals were preserved at the laboratory of standard environment of 25 ± 2 °C temperature, 55–60% relative humidity on a 12h light/dark cycle. Adequate formulated food (provided by ICDDR, B) and water supply was also maintained. Male and female mice of each five groups were used in each test. For each method, the naming of the groups was as control, standard, Group I, Group II, and Group III.

### 2.5 Ethical statement

These protocols and procedures of this study were performed, complying with the internationally accepted principles for proper use of laboratory animals by the National Institutes of Health (NIH) and the International Council for Laboratory Animal Science (ICLAS). The present study protocol was reviewed and approved by the University of Chittagong Ethics Committee.

### 2.6 Analysis of anti-nociceptive activity

#### 2.6.1 Acetic acid-induced writhing response in mice

To evaluate the peripheral analgesic activity of the crude ethanolic extracts of seeds of *H. antidysenterica*, the acetic acid-induced writhing method was applied, following the method described in Uddin *et al.* with slight modification [16]. Treatment groups: five mice in each group, a total of 25 mice were selected randomly to make five groups. Three different doses of the crude ethanolic extract were administered in this study (50, 100, and 150 mg/kg body weight). 1% acetic acid (at a dose of 0.1 mL/10 g body weight) was administered intraperitoneally to induce pain in mice. Diclofenac sodium (50 mg/kg body weight) was used as standard, and the vehicle control group received normal saline at a dose of 0.1 mL/10 gm body weight. Acetic acid was applied after 40 min of administering Diclofenac and normal saline. Five minutes after the administration of acetic acid, the total number of abdominal writhing responses were counted for 10 min and recorded. Percentage inhibition of writhing was measured as an index of analgesia by the following formula:

Inhibition (%) = [(Wc − Wt)/Wc] × 100%

Wc is the number of writhing responses in the control group, and Wt is the total number of writhing responses in the test groups.

#### 2.6.2 Formalin test

The anti-nociceptive activity of the extracts was evaluated using the formalin test described by Uddin et al. with minor modification [16]. The control group received 5% formalin. 20 μl of 5% formalin was injected into the dorsal surface of the right hind paw 60 min after administration of samples (100 and 200 mg/kg, p.o.) and Morphine (5 mg/kg, i.p.). After 30 min injection of formalin, the mice were observed, and the amount of time spent licking the injected hind paw was recorded. The first 5 min post formalin injection is referred to as the early phase and the period between 15 and 30 min as the late phase. The total time spent licking or biting the injured paw (pain behavior) was measured with a stopwatch as an indicator of pain sensation.

#### 2.6.3 Hot-plate test

The hot plate test was performed to evaluate the anti-nociceptive potentials of *H. antidysenterica* seed extracts through an established method applying minor modifications [17]. A constant temperature of 55.0 ± 0.5 °C was maintained on the surface of Eddy's hot plate throughout the experiment. All mice have undergone overnight fasting before this experiment. Mice were either treated with DMSO (p.o.) (control group) or treated with Morphine sulfate (5 mg/kg, i.p.) (standard group). The other three groups of animals Group I, Group II, Group III received *H. antidysenterica* seed extracts at a dose of 50, 100, and 150 mg/kg body weight, p.o. respectively. All the drugs and extracts were administered 30 min before the experiment. The hot plate test estimates the latencies of the reaction (forepaw licking, withdrawal of paw, or jumping). The nociceptive response from the mice was recorded with stopwatch by placing them on the heated surface at 30 min before (pretreatment) and at 30, 60, and 90 min after the treatment. Injury to paw tissue was avoided setting a maximum contact time of 20s, which was also an indication of complete analgesia [18, 19]. The analgesic effects of the extracts determined by the hot plate test were expressed using the maximal possible effect (%MPE) formula [20]:

% MPE = [(Post drug latency – Pre drug latency) / (Cut off period – Pre drug latency)] × 100

#### 2.6.4 Tail immersion test

Central anti-nociceptive activity of *H. antidysenterica* seed extracts was evaluated using previously reported methods [21]. A water bath was kept at a suitable temperature (55 ± 0.5 °C) for the tail immersion of mice. Animals for this test were also divided into five groups and marked accordingly the same as Hot plate test. Approximately 3 cm of the mice's tail end was submerged in hot water. A pain response was noted if a sudden withdrawal of the tail from the hot water occurred. The time length of tail immersion and deflection of the tail from hot water at 30 min before (pretreatment) and 30, 60, and 90 min after administration of all test samples were recorded by stopwatch. As excessive immersion of tails in hot water may cause impairments to tissues, 15s was set as maximal immersion time. An increase in immersion time indicates the potential analgesic activity of the plant extracts. The analgesic effects of the extracts determined by tail immersion test were expressed using maximal possible effect (%MPE) formula [20]:

% MPE = [(Post drug latency – Pre drug latency) / (Cut off period – Pre drug latency)] × 100

### 2.7 In silico analysis for analgesic activity

#### 2.7.1 Selection of compounds

A thorough literature review revealed several isolated compounds from different parts of the studied plant. However, compounds isolated from the seeds are selected in this study. Structures of Conimine (PubChem ID: 101686); Conessine (PubChem ID: 441082); Conarrhimin (PubChem ID: 12303820); Isoconessimine (PubChem ID: 551434); Antidysentericine (PubChem ID: 132918182); Funtudienine (PubChem ID: 102093828) were downloaded from PubChem database.

#### 2.7.2 Preparation of ligands

Before performing molecular docking, the compounds were prepared by Ligprep3.3 wizard in Schrödinger Suite-Maestro v 10.1 [22]. Individual ligands were applied with the OPLS_2005 force field, where 3D geometries were created, and proper bond orders were assigned [23]. The ligands were neutralized at pH 7.0 ± 2.0 using Epik3.1 of Schrödinger Suite. The lowest energy ring conformation of each ligand was selected for docking.

#### 2.7.3 Preparation of receptor and grid generation

Three dimensional crystal structures of cyclooxygenase-1 (COX-1, PDB ID: 2OYE) [24] and cyclooxygenase-2 (COX-2, PDB ID: 6COX) [25] was downloaded in .pdb format from the RCSB Protein Data Bank [26]. Structures of the protein targets were prepared and refined using the Protein Preparation Wizard of Schrödinger-Maestro v10.1. Charges and bond orders were assigned, and other necessary refinements were done through standard protocols [27].

A properly computed receptor grid of arranged amino acid residues of proteins is required as each ligand has a specific binding site in the receptor. Grids were created, keeping the default parameters of van der Waals scaling factor 1.00 and charge cutoff 0.25 subjected to OPLS_2005 force field. A cubic box of was generated around the active site of the receptor with a measurement of 14 Å × 14 Å × 14 Å for docking experiments [28, 29].

#### 2.7.4 Glide standard precision (SP) ligand docking

Molecular docking was performed using glide standard precision (SP) ligand docking. The docking experiment was performed as described in our previous studies [30]. The least Glide score for each ligand was considered as the best-docked mode. Finally, the best-docked poses were further analyzed by Biovia Accelrys Discovery Studio Visualizer software for 2D and 3D binding interactions with amino acid residues [31].

#### 2.7.5 Predicting pharmacokinetic parameters by SwissADME

The molecular properties of compounds play a crucial role in the selection of these agents as potential drug candidates. The pharmacokinetic properties of the compounds were predicted by using SwissADME online server (http://www.swiss.adme.ch/). The compounds were screened by Lipinski's rule of five (RO5) filter to assess their credibility as drug candidates, i.e. (i) low molecular weight (acceptable range: <500); (ii) less hydrogen bond donor (acceptable range: ≤5); (iii) less hydrogen bond acceptor (acceptable range: ≤10); (iv) high lipophilicity (expressed as log P_o/w_, acceptable range: <5); and (v) suitable molar refractivity (acceptable range: between 40 and 130). The canonical SMILES retrieved from PubChem was used through swissADME online database for this process [32, 33].

### 2.8 Statistical analysis

Data were expressed as mean ± standard error of mean (SEM) in the study. One-way analysis of variance (ANOVA) followed by Dunnett's t-test was done for statistical analysis of data employing SPSS (Statistical Package for Social Sciences) version 22.0. For statistical significance, p < 0.05 was considered.

## 3 Results

### 3.1 Acetic acid-induced writhing response

**Figure 1:**
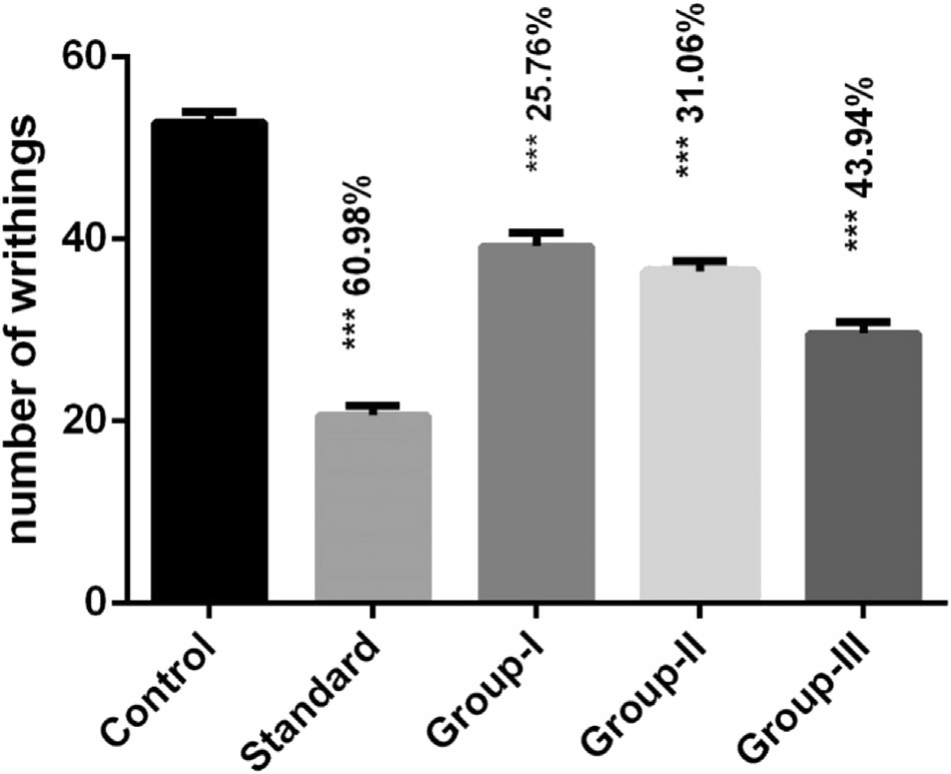
Anti-nociceptive effect of ethanolic extract of *Holarrhena antidysenterica* seeds in acetic acid-induced writhing test. Data values are presented as mean ± SEM (n = 5); % Writhing inhibition compared to control; ∗p ˂ 0.05, ∗∗p ˂ 0.01 and ∗∗∗p ˂ 0.001 compared with the control group (One way ANOVA with post hoc Dunnett's test).

Statistical evaluation of the recorded data showed that crude ethanolic extract possessed moderate peripheral analgesic activity. Among all the test groups, the crude ethanolic extract of *H. antidysenterica* seeds at 150 mg/kg body weight dose exhibited maximum peripheral analgesic activity (43.49% writhing inhibition) compared to standard (60.98% writhing inhibition). The result also indicated that lower doses also have mild to moderate peripheral analgesic activity (Figure 1).

### 3.2 Formalin test

The analgesic activity tests were carried out in the laboratory on four groups of mice by Formalin Induced Test Method. A dose-dependent anti-nociceptive relationship in formalin-induced pain was observed in the early phase. Group-II, Group-III (100, and 150 mg/kg) and Morphine (5 mg/kg) treated groups showed significant changes as compared to the control group (77.4 ± 2.89, p < 0.05; 71.2 ± 5.67, p < 0.01 and 55.4 ± 4.08, p < 0.001 respectively). In the late phase, the subcutaneous injection of formalin-induced licking and biting time was moderately decreased by pretreatment with a dose of 150 mg/kg sample (36.6 ± 5.62, p < 0.05). Morphine exerted significant anti-nociceptive activity in the late phase (20.6 ± 4.40, p < 0.001). The acquired results of the tests are presented in Table 1.

**Table 1:** Anti-nociceptive effects of *H. antidysenterica* seed extracts in formalin induced licking test.

Paw licking in mice (second)			
Test Group	Dose (mg/kg)	Early Phase	Late Phase
Control	Nil	101.0 ± 5.25	62.6 ± 5.71
Standard	5	55.4 ± 4.08^∗∗∗^ [[2], [4], [5], [6], [7], [8], [9], [10], [11], [12], [13], [14], [15], [16], [17], [18], [19], [20], [21], [22], [23], [24], [25], [26], [27], [28], [29], [30], [31], [32], [33], [34], [35], [36], [37], [38], [39], [40], [41], [42], [43], [44], [45], [46], [47], [48], [49], [50], [51], [52], [53], [54]]	20.6 ± 4.40^∗∗∗^
Group I	50	93.4 ± 6.62	57.0 ± 7.72
Group II	100	77.4 ± 2.89^∗^	43.0 ± 2.17
Group III	150	71.2 ± 5.67^∗∗^	36.6 ± 5.62^∗^

Data values are presented as mean ± SEM (n = 5); ∗p ˂ 0.05, ∗∗p ˂ 0.01 and ∗∗∗p ˂ 0.001 compared with control group (One way ANOVA with post hoc Dunnett's test).

### 3.3 Hot-plate test

A dose-dependent increment in the latency period of the heat-induced nociception was observed in the experimental mice that was much similar to the standard drug morphine (Table 2). After administration of seed extracts of *H. antidysenterica* at doses 50, 100, and 150 mg/kg body weight, the latency times were increased in all doses. Moreover, at 30 min time at dose 150 mg/kg, the pain inhibition was even stronger than the standard drug morphine. In 60 min time period the latency time and % MPE was also notably significant (p < 0.05, p < 0.01, p < 0.001 respectively for incremental doses) compared to control group. The observations of 90 min were also similar to other study periods, which show an increment of response for dose increments. Of all the doses, the lowest effect was observed in Group I at 90 min (Table 2).

**Table 2:** Anti-nociceptive effects of *H. antidysenterica* seed extracts in hot plate test.

Latency time (s) (% MPE)					
Test Group	Dose (mg/kg)	Pretreatment	30 min	60 min	90 min
Control	Nil	8.00 ± 0.89	8.40 ± 0.40	8.80 ± 0.58	8.40 ± 0.75
Standard	5	10.80 ± 0.37	14.80 ± 0.86^∗∗∗^(43.48)	16.60 ± 0.51^∗∗∗^(63.04)	12.40 ± 0.51^∗∗^ (17.39)
Group I	50	9.80 ± 0.97	11.60 ± 0.51^∗^ (17.65)	11.20 ± 0.86^∗^ (13.73)	10.80 ± 0.37 (9.80)
Group II	100	10.40 ± 0.51	13.20 ± 1.36^∗∗^ (29.17)	12.20 ± 0.58^∗∗^ (18.75)	11.60 ± 1.08^∗^ (12.50)
Group III	150	10.80 ± 0.37	17.00 ± 1.27^∗∗∗^(67.39)	13.60 ± 0.93^∗∗∗^ (30.43)	13.20 ± 1.02^∗∗∗^(26.09)

Data values are presented as mean ± SEM (n = 5); MPE, Maximum possible effect; Numbers in parentheses indicates %MPE compared to control; ∗p ˂ 0.05, ∗∗p ˂ 0.01 and ∗∗∗p ˂ 0.001 compared with control group (One way ANOVA with post hoc Dunnett's test).

### 3.4 Tail immersion test

In the tail-immersion test, seed extracts of *H. antidysenterica* and morphine showed a significant reduction in pain perception at all doses induced by hot water, which is a notable indication of dose-dependent anti-nociceptive activity. The %MPE values of *H. antidysenterica* seed extracts and morphine were significant (p < 0.001 in 30 min and p < 0.01 in 60 min for both Group I and Group II, and p < 0.001 for morphine). The anti-nociceptive activity was higher in the standard group than the experimental doses. In contrast, all the doses of seed extracts showed increased latency time compared to the control group (Table 3).

**Table 3:** Anti-nociceptive effects of *H. antidysenterica* seed extracts in the tail immersion test.

Latency time (s) (% MPE)					
Test Group	Dose (mg/kg)	Pretreatment	30 min	60 min	90 min
Control	Nil	2.00 ± 0.32	4.60 ± 0.60	5.00 ± 0.45	9.80 ± 1.07
Standard	5	3.00 ± 0.32	13.20 ± 0.66^∗∗∗^(85.00)	12.20 ± 0.73^∗∗∗^(76.67)	11.80 ± 0.58 (73.33)
Group I	50	2.00 ± 0.45	3.80 ± 0.73 (13.85)	8.20 ± 1.71 (47.69)	10.00 ± 0.55 (61.54)
Group II	100	2.40 ± 0.51	9.60 ± 0.93^∗∗∗^(57.14)	11.00 ± 0.95^∗∗^(68.25)	10.40 ± 0.68 (63.49)
Group III	150	2.40 ± 0.51	11.20 ± 0.73^∗∗∗^(69.84)	10.00 ± 1.00^∗∗^(60.32)	9.40 ± 1.02 (55.56)

Data values are presented as mean ± SEM (n = 5); MPE, Maximum possible effect; Numbers in parentheses indicates %MPE compared to control; ∗p ˂ 0.05, ∗∗p ˂ 0.01 and ∗∗∗p ˂ 0.001 compared with control group (One way ANOVA with post hoc Dunnett's test).

### 3.5 Molecular docking study

**Figure 2:**
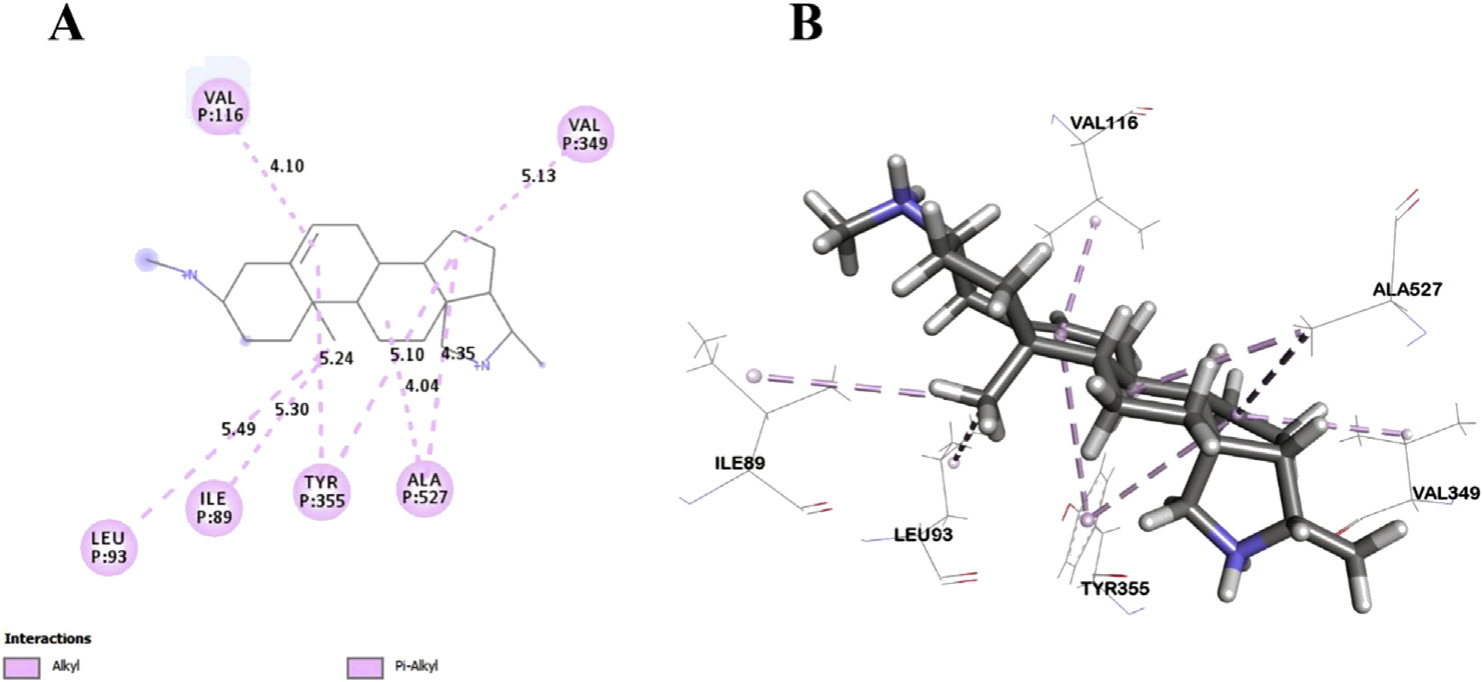
Best ranked pose of Conimine; 2D (A) and 3D (B) interactions in the binding pocket of COX-1 (PDB ID: 2OYE).

**Figure 3:**
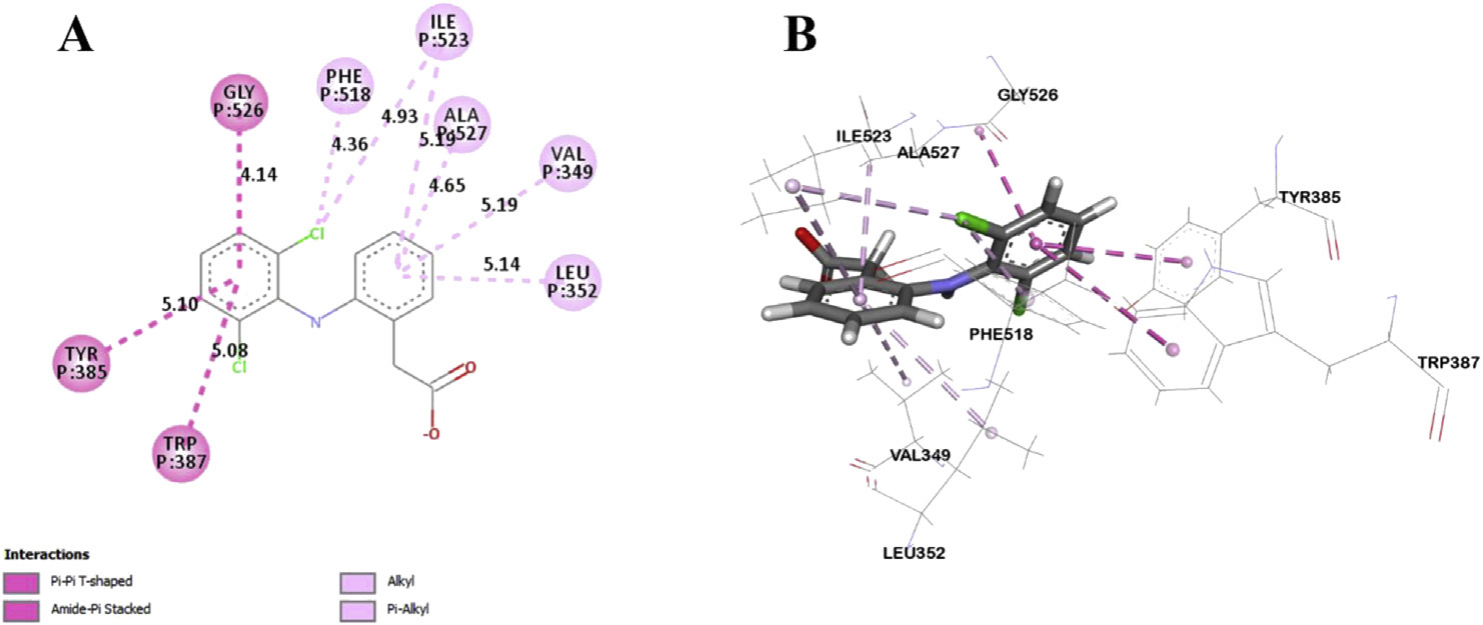
Best ranked pose of Diclofenac; 2D (A) and 3D (B) interactions in the binding pocket of COX-1 (PDB ID: 2OYE).

**Figure 4:**
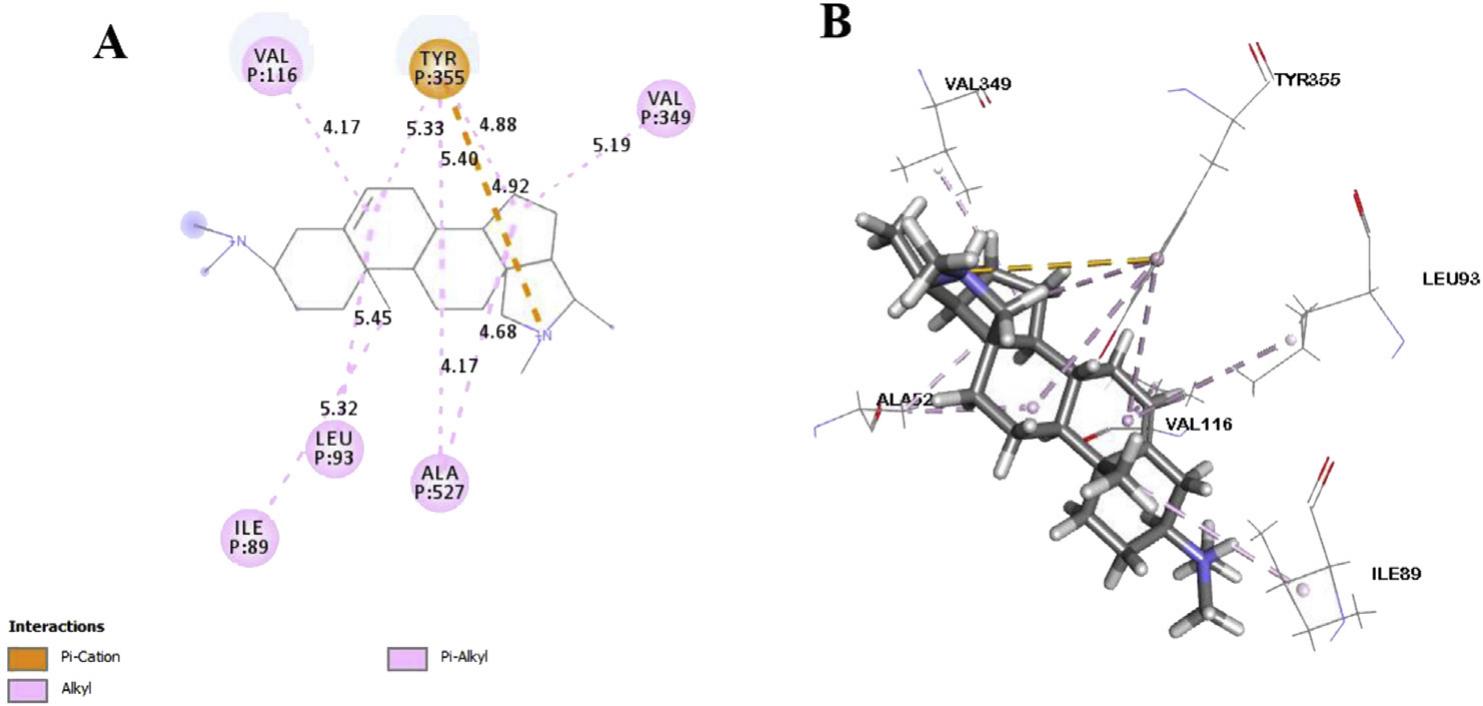
Best ranked pose of Conarrhimin; 2D (A) and 3D (B) interactions in the binding pocket of COX-1 (PDB ID: 2OYE).

**Figure 5:**
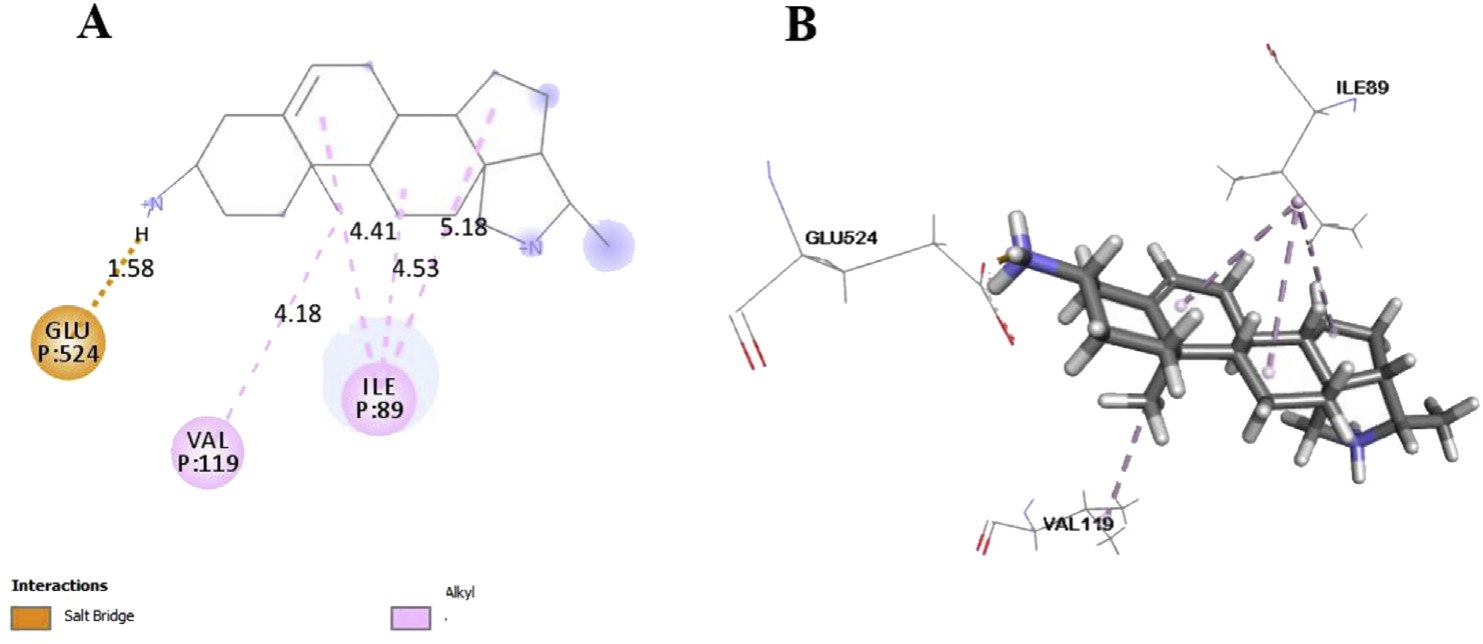
Best ranked pose of Conessine; 2D (A) and 3D (B) interactions in the binding pocket of COX-1 (PDB ID: 2OYE).

**Figure 6:**
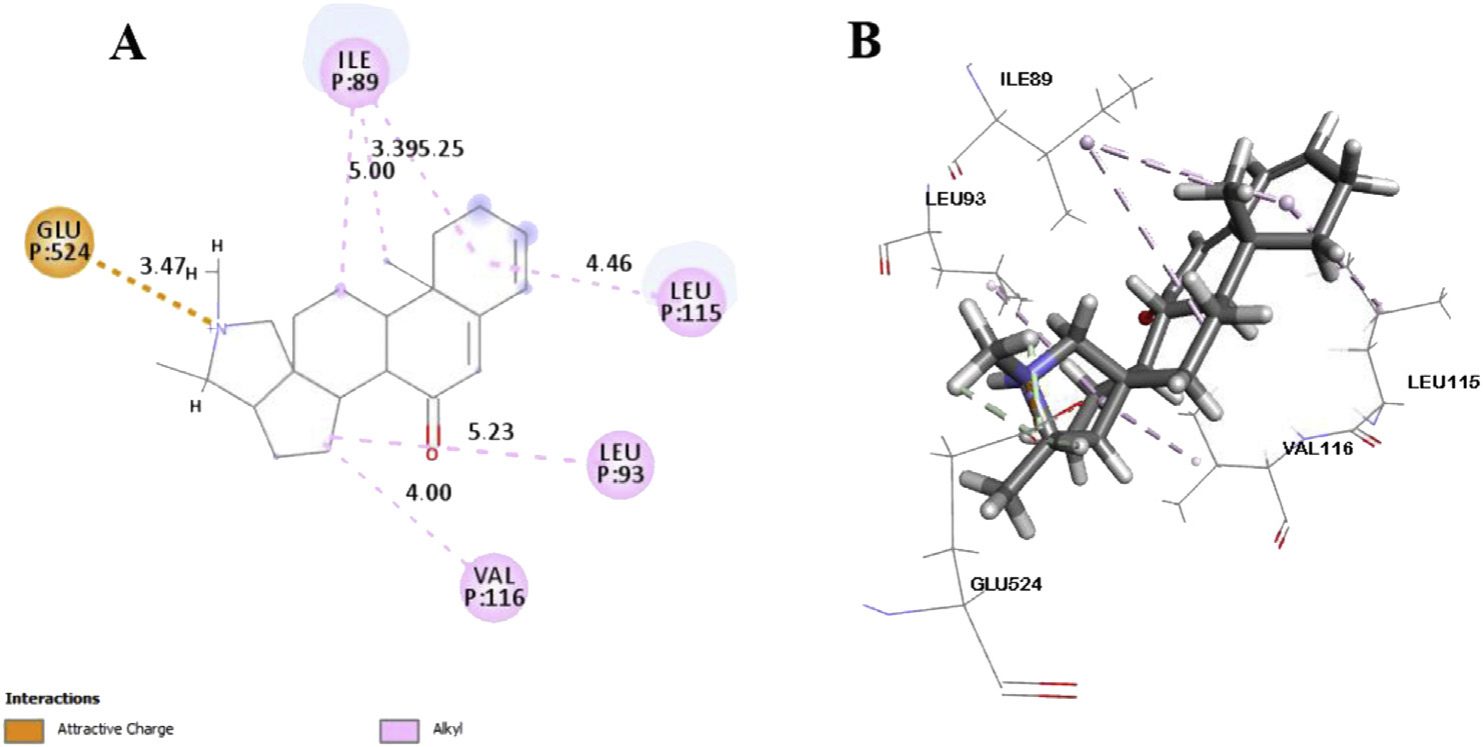
Best ranked pose of Funtudienine; 2D (A) and 3D (B) interactions in the binding pocket of COX-1 (PDB ID: 2OYE).

In this study, six major compounds of *H. antidysenterica* were docked against two enzymes, namely COX-1 (PDB: 2OYE) and COX-2 (PDB: 6COX). Results against COX-1 showed that Conimine has the best binding affinity against the COX-1 enzyme with the highest docking score (-4.93 kcal/mol) followed by Conarrhimin (-4.815 kcal/mol), Conessine (-4.773 kcal/mol) and Funtudienine (-4.517 kcal/mol). However, two compounds Antidysentericine and Isoconessimine, didn't bind with the COX-1 enzyme. On the other hand, no compounds showed binding scores towards the COX-2 enzyme; hence can be considered not having an affinity towards COX-2. The result of the docking study is shown in Table 4. The interactions of best-docked molecule (Conimine) and standard drug Diclofenac with amino acid residues of COX-1 are presented in Figures 2 and 3 respectively. Molecular interactions of other molecules with this receptor are shown in Figures 4, 5, and 6.

**Table 4:** Molecular docking score and bond interactions analysis of the best docked ligands and celecoxib against COX-1.

Molecule	Docking score (kcal/mol)	Hydrogen bonds interactions		Hydrophobic interactions	
		Residue (interaction type)	Distance (Å)	Residue (interaction type)	Distance (Å)
Conimine	-4.930			TYR355 (Pi-Alkyl)	5.24
				TYR355 (Pi-Alkyl)	5.10
				LEU93 (Alkyl)	5.49
				ILE89 (Alkyl)	5.30
				ALA527 (Alkyl)	4.04
				ALA527 (Alkyl)	4.35
				VAL349 (Alkyl)	5.13
				VAL116 (Alkyl)	4.10

Conarrhimin	-4.815	GLU524 (Salt Bridge)	1.58	VAL119 (Alkyl)	4.18
				ILE89 (Alkyl)	4.41
				ILE89 (Alkyl)	4.53
				ILE89 (Alkyl)	5.18

Conessine	-4.773	TYR355 (Pi-Cation)	4.92	VAL116 (Alkyl)	4.17
				VAL349 (Alkyl)	5.19
				ALA527 (Alkyl)	4.17
				ALA527 (Alkyl)	4.68
				LEU93 (Alkyl)	5.45
				ILE89 (Alkyl)	5.32
				TYR355 (Pi-Alkyl)	5.33
				TYR355 (Pi-Alkyl)	5.40
				TYR355 (Pi-Alkyl)	4.88

Funtudienine	-4.517	GLU524 (Attractive Charge)	3.47	VAL116 (Alkyl)	4.00
				LEU93 (Alkyl)	5.23
				LEU115 (Alkyl)	4.46
				ILE89 (Alkyl)	5.00
				ILE89 (Alkyl)	3.39
				ILE89 (Alkyl)	5.25

Diclofenac	-8.207			GLY526 (Pi-Pi T-shaped)	4.14
				TYR385 (Pi-Pi T-shaped)	5.10
				TRP387 (Pi-Pi T-shaped)	5.08
				ILE523 (Alkyl)	4.93
				PHE518 (Pi-Alkyl)	4.36
				ILE523 (Pi-Alkyl)	5.19
				ALA527 (Pi-Alkyl)	4.65
				VAL349 (Pi-Alkyl)	5.19
				LEU352 (Pi-Alkyl)	5.14

Post docking analysis of the compounds indicated several binding interactions between the amino acid residues of the target enzymes and the ligands. Conimine showed eight interactions with COX-1 enzyme through hydrophobic interactions involving TYR355 (two interactions), LEU93, ILE89, ALA527 (two interactions), VAL349 and VAL116. Conarrhimin showed both hydrogen bond with GLU524 and four hydrophobic interactions with VAL119, and ILE89 (three interactions). The other molecule Funtudienine also had both hydrogen bond with GLU524 and six hydrophobic interactions with VAL116, LEU93, LEU115, and ILE89 (three interactions).

### 3.6 Pharmacokinetic properties

Compounds should be screened if they possess compatible pharmacokinetic properties with the biological system before considering them as potential drug candidates. Lipinski's rule of five is an effective tool to screen drug-likeness of compounds. The properties are: (i) molecular weight <500; (ii) H-bond acceptors ≤10; (iii) H-bond donors ≤5; (iv) Lipophilicity <5; and (v) molar refractivity between 40 and 130. All of the four compounds were found suitable as they satisfied the Lipinski's rule of five and Veber's rule which is a clear indication that all four compounds could be suitable as new drug development (Table 5).

**Table 5:** Physicochemical properties of the compounds by SwissADME.

Compounds	PID	Lipinski rules					Lipinski's violations	Veber rules	
		MW	HBA	HBD	Log P	MR		NRB	TPSA
Rule	-	<500	<5	≤10	≤5	40–130	≤1	≤10	≤140 Å^2^
Conimine	101686	328.53	2	2	3.84	105.82	0	1	24.06
Conarrhimin	12303820	314.51	2	2	3.48	100.92	0	0	38.05
Conessine	441082	356.59	2	0	4.35	115.63	0	1	6.48
Funtudienine	102093828	325.49	2	0	3.85	102.84	0	0	20.31

PID = Pubchem ID; MW = Molecular weight; g/mol; HBD = Hydrogen bond donor; HBA = Hydrogen bond acceptor; Log P= Lipophilicity; MR = Molar refractivity; NRB = Number of rotatable bond; TPSA = Topological polar surface area.

## 4 Discussion

Acetic acid-induced writhing response technique is a widely used method to evaluate the peripheral analgesic activity of any plant part, where acetic acid the key inducer of pain in an animal model [34]. It has been believed that the response is induced by peritoneal mast cells and prostaglandin pathways [35, 36]. When acetic acid is administered intraperitoneally, it enhances the release of some inflammatory mediators, including substance P, bradykinin, serotonin, histamine, and prostaglandins. The release of these inflammatory mediators is responsible for further abdominal constrictions or feelings of pain [37]. Deraedt et al. also explained the elevated quantification of prostaglandins in the peritoneal exudates after intraperitoneal injection of acetic acid [38]. They observed an excessive amount of prostaglandins PGE2α e PGF2α immediately after 30 min of acetic acid injection. Moreover, it was found that intraperitoneal administration of acetic acid also induces the liberation of some sympathetic nervous system mediators [39, 40]. Jiang et al. also described that lipoxygenase enzyme level increases at peritoneal fluid after intraperitoneal acetic acid injection [41]. So, it may be assumed that the mechanism of the peripheral analgesic activity of the ethanolic extract of the seed may act involving the inhibition of biosynthesis or release of these mediators.

The formalin test involves two distinct phases in its response. In the first 5 min after the formalin injection, the first phase occurs, which is referred to as the early phase (neurogenic nociceptive response). The second phase (inflammatory nociceptive response) starts between 15 to 30 min after formalin injection. Studies have shown that analgesic drugs (opioids or centrally acting analgesics) appear to suppress pain behaviors of both phases. On the other hand, non-steroidal anti-inflammatory drugs (NSAIDs) are effective only in the second phase [42]. Additionally, formalin injection produces signs of inflammation such as redness and swelling of the injected paw in the inflammatory phase due to the release of histamine, bradykinin, serotonin, and substance P-type chemical mediators [43]. Our observed result showed that the extract reduced both phases as compared with the control, which is a resemblance to the observation of Onasanwo and Elegbe [44]. Therefore inhibition in both the early and late phases in formalin-induced licking test as well as inhibiting the writhing in acetic acid-induced test indicates the potentiality of both central and peripheral anti-nociceptive activity of the seed extract of *H. antidysenterica*.

Tail immersion and hot plate are the two commonly used methods for evaluating central analgesic activity in animal models that use thermal stimuli as pain inducer. These methods emphasize the changes above the spinal cord level, which are an effective illustration of centrally mediated anti-nociceptive responses [45]. The methods are preferable for their high selectivity towards opioid-derived analgesics [46]. The seed extracts of *H. antidysenterica* showed significant anti-nociceptive activity in both models in a dose-dependent manner. A significant dose-dependent increase in latency time in the hot plate method is suggestive of the central anti-nociceptive activity of the extracts. This method induces pain through a supra-spinal reflex involving μ1, κ3, δ1, σ2 opioid receptors [47, 48, 49]. Inhibition of nociceptive activity indicates a possible inhibition or modification of pain induction through this pathway. Also, the results of the tail immersion test were supportive of the outcomes of the hot plate method. This method is selective for central analgesia as peripherally acting agents do not show effectivity in such type of thermal stimuli [50]. The μ2, κ1, and δ2 opioid receptors are involved in nociception via spinal reflexes, which can be measured through the tail immersion method [47, 48, 49]. A dose-dependent increase in anti-nociceptive activity was also shown in this method. Outcomes from both the methods are suggestive of spinal and supra-spinal receptors mediated anti-nociceptive activities of *H. antidysenterica* seed extracts.

Computational techniques are the most advanced approach in the theoretical prediction of ligand-target interactions, and it works as a validation for the observed pharmacological activities of plant extracts. Also, to correctly analyze the binding modes of active compounds against the targeted receptors, it plays a key role [51, 52]. The correct identification of binding modes and affinities can be accurately predicted by this comprehensive protocol using the X-ray crystallographic structure of proteins [53]. A set of isolated compounds from *H. antidysenterica* was thus computationally analyzed against cyclooxygenase-1 and cyclooxygenase-2 enzymes. The docking scores and binding interactions against COX-1 are reported in Table 4. Of all the compounds, Conimine showed the highest docking score (-4.93 kcal/mol) against COX-1 compared to the standard drug Diclofenac (-8.207 kcal/mol). This finding is suggestive that the compounds in seeds might have mild peripheral analgesic activities. Along with docking score, bond interactions between ligand and amino acid residues of receptor is a pivotal point for optimum pharmacological activity. Thus, visual evaluation of the best docked compound for 2D and 3D interactions with amino acid residues of the COX-1 receptor revealed that most of the bonds are hydrophobic with small distances (Table 4) indicating strong binding interactions. However, none of these compounds showed docking score against COX-2 receptor aiding into conclusion that the phytochemicals from the seeds of this plant is selective towards COX-1 receptor.

Along with their docking scores, the compounds were also checked for their pharmacokinetic parameters as many of the drug candidates fail due to their poor bioavailability and poor oral absorption that should be screened before any clinical trials. Also, the pharmacokinetic properties mostly depend on chemical descriptors of the molecules [54]. Considering this point of view, the pharmacokinetic properties (ADME: absorption, distribution, metabolism, elimination) of the selected compounds based on Lipinski's rule of five were screened from the scores got from SwissADME online server. Lipinski's rule of five (RO5) critically applies some criteria for selecting a drug suitable for oral administration. According to Lipinski's RO5, an ideal drug candidate should have a molecular weight of <500, Lipophilicity value, LogP ≤5, a number of hydrogen bond acceptor number <5, and the number of hydrogen bond donor ≤10 of which not more than one rule should be violated. The current study exhibited that none of the four docked compounds violated these rules, indicating good oral bioavailability of these molecules (Table 5). Additionally, experimental results from Veber et al. suggested that a compound should have the number of rotatable bonds (NRB) ≤ 10 and topological polar surface area (TPSA) value ≤140 Å^2^ to be an ideal drug candidate. Here NRB expresses the molecular flexibility of a molecule for suitable drugs, and TPSA is involved in passive molecular transport of drugs through membranes [54]. All the four compounds satisfied the descriptors of Veber rule, which indicates that these compounds can easily be transported through membranes and can be considered as potential drug molecules with receptor-based optimization.

## 5 Conclusion

The observed data from this study revealed that seeds of *H. antidysenterica* possess the significant potentiality of having central and peripheral anti-nociceptive activity in different *in-vivo* models. The result of these *in-vivo* tests encourages us to perform further motor performance of the animals to ascertain the potentiality of central and peripheral anti-nociceptive activity. Moreover, these results also create new scope of studies to perform suitable chromatography techniques to find out the possible phytomolecules responsible for the observed anti-nociceptive activity. Also, in the molecular docking study, the compounds isolated from seeds of this plant showed a moderate affinity towards the COX-1 enzyme. Thus, it can be concluded that the suggestive compounds from the *in-silico test* could be a good source for the development of new anti-nociceptive and anti-inflammatory agents, which support the experimental results demanding further comprehensive study to reveal their in-depth molecular mechanism of action in animal models.

## Declarations

### Author contribution statement

N. M. Mahmudul Alam Bhuiya: Conceived and designed the experiments; Performed the experiments; Analyzed and interpreted the data; Wrote the paper.

Md. Mahbubur Rahman Bhuiyan and U.J. Nahar: Performed the experiments.

N. Hasan: Performed the experiments; Analyzed and interpreted the data; Wrote the paper.

### Funding statement

This research did not receive any specific grant from funding agencies in the public, commercial, or not-for-profit sectors.

### Competing interest statement

The authors declare no conflict of interest.

### Additional information

No additional information is available for this paper.
